# Study on the Fracture Toughness of Softwood and Hardwood Estimated by Boundary Effect Model

**DOI:** 10.3390/ma15114039

**Published:** 2022-06-06

**Authors:** Hong-Mei Ji, Xiao-Na Liu, Xiao-Wu Li

**Affiliations:** Department of Materials Physics and Chemistry, School of Material Science and Engineering and Key Laboratory for Anisotropy and Texture of Materials, Ministry of Education, Northeastern University, Shenyang 110819, China; jihongmei@mail.neu.edu.cn (H.-M.J.); lxn19960904@163.com (X.-N.L.)

**Keywords:** softwood, hardwood, boundary effect model, tensile strength, fracture toughness

## Abstract

The tensile strength and fracture toughness of softwood and hardwood are measured by the Boundary Effect Model (BEM). The experimental results of single-edge notched three-point bending tests indicate that the BEM is an appropriate method to estimate the fracture toughness of the present fibrous and porous woods. In softwood with alternating earlywood and latewood layers, the variation in the volume percentage of different layers in a small range has no obvious influence on the mechanical properties of the materials. In contrast, the hardwood presents much higher tensile strength and fracture toughness simultaneously due to its complicated structure with crossed arrangement of the fibers and rays and big vessels diffused in the fibers. The present research findings are expected to provide a fundamental insight into the design of high-performance bionic materials with a highly fibrous and porous structure.

## 1. Introduction

Trees, one of the most common plants in nature, are playing an important role in the production and daily life of human beings, since they not only provide fresh air, but also provide a renewable and sustainable resource [1,2,3]. They have survived successfully on earth for hundreds of millions of years, and there are more than 3 trillion mature trees on earth, covering over 30% of the land. As one of the most important products of trees, wood has been ubiquitously used for construction, furniture, and tools for thousands of years, because of several physical properties: it is lightweight with a high strength-to-weight ratio, tractable, and has a low environmental impact [4,5,6,7].

The main building block of wood is cellulose (40–45 wt.%), which has exceptional intrinsic mechanical properties, i.e., it has a high flexural modulus (~150 GPa), a high theoretical tensile strength (~1.6–7.7 GPa), and a high specific strength (1.0–5.1 GPa⋅cm^3^⋅g^−^^1^) due to the low density [8,9,10,11]. Different woods present variations in microstructure even though they are all mainly composed of cellulose. Softwoods are mainly constructed by one cell type named axial tracheids (~95%) that are oriented mostly in the axial direction [12]. The cellular elements along the radial material direction lead to a periodic pattern of concentric rings, named annual growth rings. In softwood, each growth ring consists of earlywood in light color and latewood in dark color with smaller cell cavities. Thus, the microstructure of softwood is relatively simple. Although the mechanical properties of softwood have drawn some attention [12,13,14,15], there is limited research concerning the influence of the growth ring on the fracture behavior of softwood. In contrast, hardwoods normally present a more complex structure with different types of cells. Specifically, fibers and axial parenchyma are disrupted by vessels with a relatively large diameter [16,17,18,19]. However, there is limited research regarding the influence of such differences in microstructures of softwood and hardwood on their mechanical behaviors.

Strength and fracture toughness are important parameters to evaluate the mechanical properties, and they are a material constant used only for homogeneous materials. Normally this is not possible for bio-samples. That is, a composite model is needed to extrapolate useful material constants from “non-constant test results”. Based on the method of non-linear elastic fracture mechanics, Hu’s research group proposed the Boundary Effect Model (BEM) method that links the tensile strength *f*_t_ and *K*_IC_ [20,21,22,23]. The BEM has successfully measured the *f*_t_ and *K*_IC_ of bamboo with values of 114.7 MPa and 7.96 MPa⋅m^1/2^ [24]. Therefore, in the present work, the *Pinus sylvestris var. mongolica Litv* (PSV) and *Black Walnut* (BW) were selected as the target experimental materials, and the BEM solutions were used to explore their fracture behavior. It is hoped that the influence of the growth ring on the fracture behavior of softwoods, and the difference in the mechanical properties of the softwood and hardwood, can be clearly demonstrated by quantitatively calculating the fracture toughness *K*_IC_, thus providing a novel guideline for improving the fracture properties of porous and fibrous materials.

## 2. Materials and Methods

**Figure 1:**
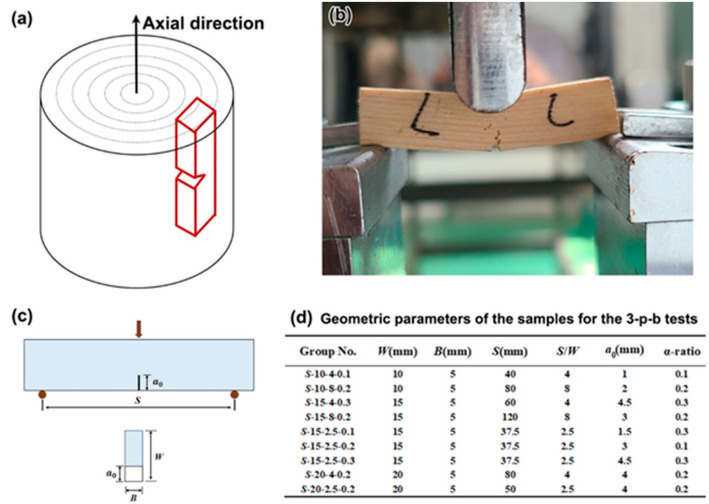
Schematic diagram for the preparation of samples (**a**) and the actual loading device (**b**) for 3-p-b tests, and schematic diagram of the dimension of samples (**c**), together with the geometric parameters (listed in Table) (**d**) for the 3-p-b tests.

The *Pinus sylvestris var. mongolica Litv* and *Black walnut* are two of the most common types of trees on earth, which are commonly used for furniture. In the present work, the microstructure on the transversal and longitudinal (parallel to the axil direction of the wood, as shown in Figure 1a) planes of PSV and BW were observed by optical microscope (OM) and scanning electron microscope (SEM). In order to calculate the fracture toughness by BEM, single-edge notched three-point bending (3-p-b) tests were performed on these two types of woods, which were carried out by an AG-Xplus machine at a loading rate of 0.1 mm/min (Figure 1b). Figure 1a is the schematic diagram of the sampling direction for single-edge notched 3-p-b tests, and Figure 1c is the schematic diagram of the dimension of samples, where *S*, *B* and *W* represent the sample thickness, height, and span, respectively, and *a*_0_ is the initial notch depth. As listed in the table in Figure 1d, nine groups of single-edge notched samples (a total of 73 samples) were prepared with different *W*, *S*/*W* and *α*-ratio (*α* = *a*_0_/*W*), and there are eight or nine samples in each group. The single-edge notch was prepared with a diamond wire cutting machine with a wire of 0.35 mm in diameter. After 3-p-b tests, the fracture surfaces of representative failed samples in each group were observed by SEM.

## 3. Results and Discussion

### 3.1. Microstructures

**Figure 2:**
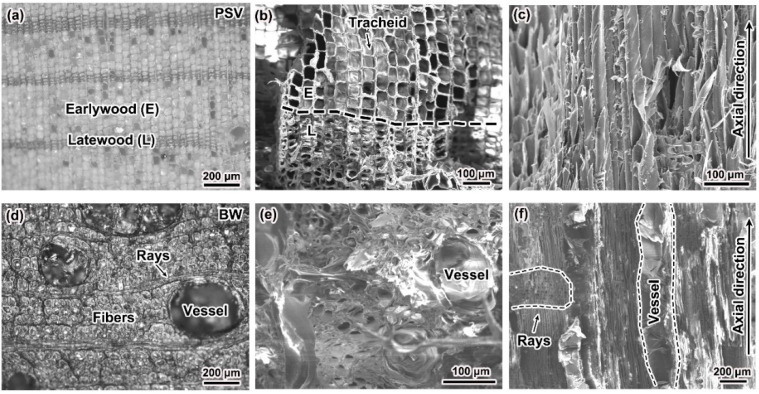
Microstructures on the transversal plane (**a**,**b**) and longitudinal plane (**c**) of PSV, and transversal plane (**d**,**e**) and longitudinal plane (**f**) of BW.

Figure 2 gives the microstructures on different planes of PSV and BW. Clearly, the growth rings are clearly seen on the transversal plane of PSV (Figure 2a), i.e., the light colour region and dark colour region are arranged alternatively. In each growth ring, the light colour region is called earlywood, and the dark colour region is named latewood. From Figure 2b, it can be observed that the PSV is mainly consisted of tracheid. The cell cavities of the tracheid show a roughly square shape in earlywood, but a more flat shape in latewood. Furthermore, the volume of the cell cavities in earlywood is much larger than that in latewood (Figure 2b). The tracheid is arranged along the axial direction of the wood (Figure 2c).

In contrast, the microstructure observed on the transversal plane of BW is more complicated than that of PSV. It is interesting to note that the BW is mainly constructed by fibers separated by long rays, and large pores named vessels are dispersed among the fibers (Figure 2d). The cell cavities of the fibers are rough round, and the cell wall is much thicker than that of the tracheid in PSV (Figure 2e). The fibers are also parallel to the axial direction of the wood, but the rays are perpendicular to the axial direction, as shown in Figure 2f. 

Based on the above results, it is clear that the PSV is a typical type of softwood, and BW is a type of hardwood. According to the results of mercury injection experiment (AutoPore lv9500), the average pore size and average porosity of PSV are around 39.5 μm and 66.0%, and of BW are 27.1 μm and 51.4%, respectively. So, these two types of wood are highly porous, but the BW is a little denser than PSV.

### 3.2. Mechanical Properties

**Figure 3:**
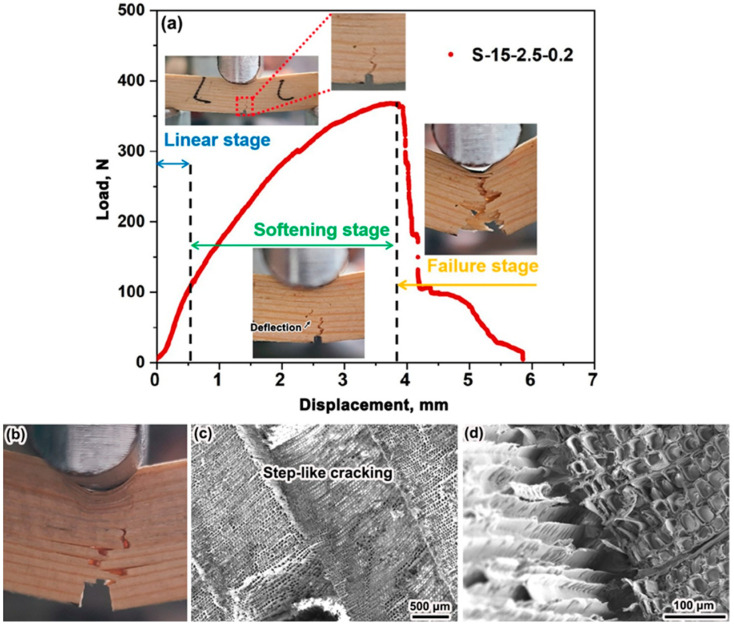
Simplified three-stage quasi-brittle fracture process (**a**), the side view under testing (**b**), and detailed morphologies of the fracture surface (**c**,**d**) of the PSV sample.

Figure 3 gives a representative load vs. displacement curve of the PSV sample under single-edge notched 3-p-b test. It is interesting to note that the curve has deflection softening stages before reaching the *P*_max_ in contrast to the initial slopes. Clearly, the fracture process can be divided into three stages, including linear stage, softening stage, and failure stage. The changes in the side surface of a sample during the 3-p-t test is shown in Figure 3a, which indicates that the crack is initiated at the notch, and it penetrates into the latewood and earlywood with minor deflection at the linear stage. In such a stage, the deformation increases due to the elastic strain of tracheid, and thus the curve keeps linearly. With the load increasing, the crack begins to deflect along the interfaces between the earlywood and latewood at the softening stage. Such an interface cracking eventually leads to the failure of the sample, which is the main failure mode of PSV (Figure 3b). The step-like cracking among the earlywood and latewood can be clearly observed on the detailed morphologies of the fracture surface in Figure 3c. The interface cracking is mainly attributed to the peeled tracheid (Figure 3d). 

In BEM solutions, the ft and *K*_IC_ of the materials will be obtained by using the peak load *P*_max_, the specimen geometry, and average grain size *G* in single-edge 3-p-b tests. The major characteristic of this method is that the *P*_max_ is proportional to the equivalent area *A*_e_, and the proportional coefficient is exactly *f*_t_. Therefore, according to the BEM, *f*_t_ and *K*_IC_ can be calculated as follows [24],

(1) $$p_{\max}=f_{t}\cdot A_{e}\left(W,a_{0},G\right)=f_{t}\cdot \frac{\left(W-a_{0}\right)\cdot \left(W-a_{0}+3\cdot G\right)}{1.5\cdot \left(\frac{S}{B}\right)\cdot \sqrt{1+\frac{a_{e}}{3\cdot G}}}$$

(2) $$K_{I\;C}=2f_{t}\cdot \sqrt{3\cdot G}$$

where *A*_e_ is the equivalent crack length that is decided by *a*_0_ and *α*, and its value can be obtained based on the appropriate formulae [20,25]. The determine of the average grain size *G* is a very important step. Normally, the *G* is the dimension of the basic building blocks of materials [21]. For example, the average grain size is identified as the diameter of fiber bundle of bamboo–fiber composites [24], and is confirmed as the thickness of bricks of large-scale brick-mortar structure [26]. In this way, the average grain size of wood should be the average diameter of the tracheid, and thus the *G* is measured to be ~39.50 μm. 

**Figure 4:**
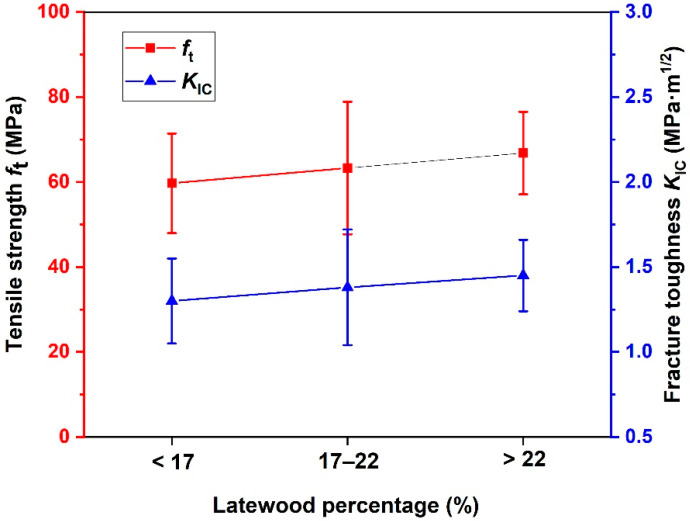
Distribution of the tensile strength and fracture toughness with different volume percentages of the latewood of PSV.

The PSV samples are divided into three groups based on the volume percentage of the latewood. The normal distribution is used as a statistical model to study the experimental scatters (see Figure S1), and Figure 4 gives the distribution of the *f*_t_ and *K*_IC_ of samples with different volume percentages of latewood. Obviously, the *f*_t_ and *K*_IC_ roughly increases whilst the volume percentage of the latewood increases, but not greatly. The tensile strength is 59.7 MPa for samples with a volume percentage of the latewood lower than 17%, 63.3 MPa for samples with a volume percentage of the latewood 17–22%, and 66.8 MPa for samples with a volume percentage of the latewood higher than 22%. The corresponding fracture toughness of PSV is 1.30, 1.38, and 1.45 MPa·m^1/2^, respectively. The ANOVA results show that at the 0.05 level, the differences in the *f*_t_ and *K*_IC_ among the three groups are not statistically significant. This illustrates that the volume percentage of the latewood has little influence on the *f*_t_ and *K*_IC_, which may be a result of the variation of the volume percentage of the latewood being small.

**Figure 5:**
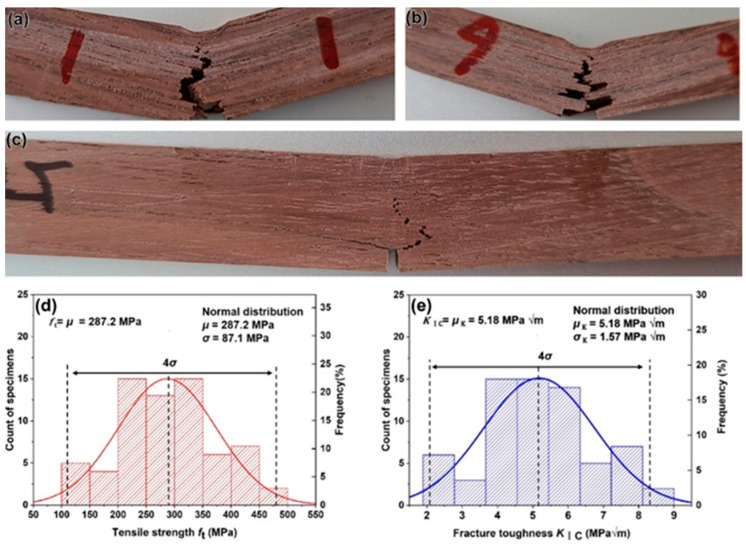
(**a**–**c**) Side views of the failed samples, and the normal distribution of the tensile strength (**d**) and fracture toughness (**e**) of BW.

**Figure 6:**
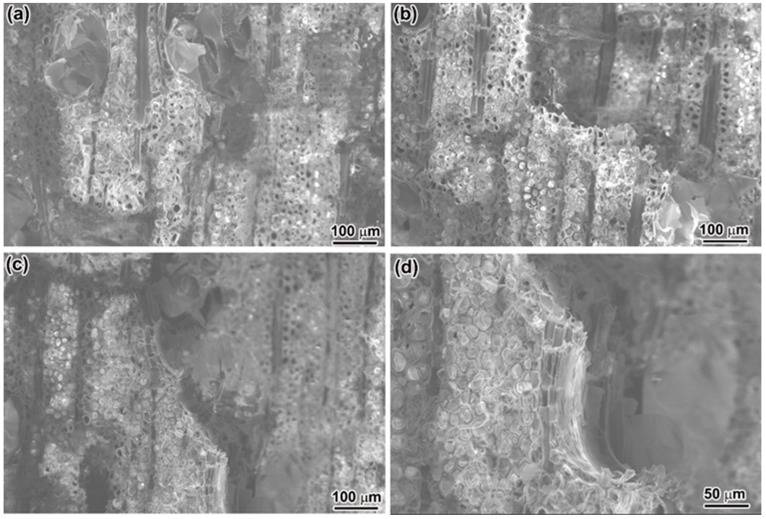
SEM images of fractured vessels (**a**,**b**), cracks propagate from one vessel to the adjacent vessel (**c**), and the magnified images of one fractured vessel (**d**) in failed BW samples.

In contrast to a side view of the failed samples of PSV, the crack paths in the BW samples vary among different samples, as their microstructures are different ahead of the notches, as shown in Figure 5a–c. The tensile strength and fracture toughness estimated by BEM solutions are given in Figure 5d,e, respectively. The tensile strength reaches a higher value of ~287.2 MPa, as well as the fracture toughness with a value of 5.18 MPa·m^1/2^. Here, the average grain size *G* of BW is the average diameter of the fibers with a value of 27.51 μm. Such higher values of tensile strength and fracture toughness have a closer relationship with their complicated microstructure. Figure 6 shows the detailed morphologies of the fracture surfaces of BW samples, on which the fracture of the vessels can be clearly seen (Figure 6a,b), and the cracks tend to propagate from one vessel to the adjacent vessel (Figure 6c). Such a big pore ahead of the crack tip could relieve the stress concentration and can be attributed to the crack deflection (Figure 6c,d). Furthermore, the crossed arrangement of the fibers and rays provides additional resistance to crack propagation, which has been improved in the crossed-lamellar structure in mollusk shells [27,28,29,30].

**Figure 7:**
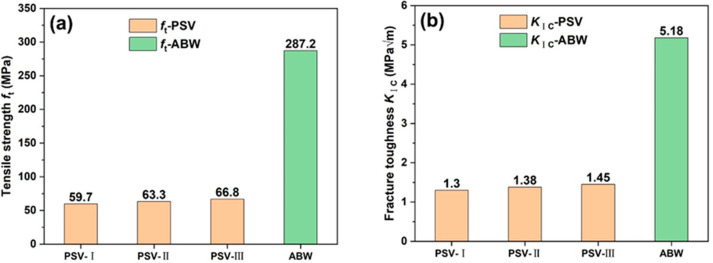
Comparison of the tensile strength (**a**) and fracture toughness (**b**) of PSV and BW.

The tensile strength is arranged from 51.0 to 120.7 MPa for hardwood and from 45.4–117.7 MPa for softwood, and the fracture toughness of wood is arranged from 2.05 to 5.17 MPa·m^1/2^ [31]. The estimated values of *f*_t_ and *K*_IC_ of the PSV and BW are roughly within such ranges. Therefore, the BEM is an appropriate method to estimate the fracture toughness of the present woods. To summarize, the PSV and BW present very different microstructures, i.e., the PSV is a typical type of softwood with an alternative arrangement of earlywood and latewood, while the BW is a type of hardwood with a complicated arrangement of fibers, rays, and vessels. The porosity of BW is only a little lower than that of PSV, but the tensile strength and fracture toughness of BW are several times higher than those of PSV, as given in Figure 7a,b. It is indicated that changing the volume percentage of the thick fibers in a small range has no obvious influence on the mechanical properties of the layered structure with alternating arrangements of thick and thin fibers. In contrast, the crossed arrangement of fibers can significantly improve the resistance to the fracture deformation of materials.

## 4. Conclusions

The BEM method can be adopted to effectively estimate the *f*_t_ and *K*_IC_ of PSV (softwood) and BW (hardwood). With the increase in the volume percentage of the latewood, the *f*_t_ and *K*_IC_ of the softwood roughly increases concurrently. The tensile strength and fracture toughness of the softwood are arranged from 59.7 to 66.8 MPa, and from 1.30 to 1.45 MPa·m^1/2^, respectively, while they reach higher values of about 287.2 MPa and 5.18 MPa·m^1/2^ in hardwood. The ability of hardwood to resist fracture can be effectively improved due to their complicated structure, showing a much higher tensile strength and fracture toughness than those of softwood.

## Data Availability

The data presented in this study are available on request from the corresponding author.

## Supplementary Materials

The following supporting information can be downloaded at: https://www.mdpi.com/article/10.3390/ma15114039/s1, Figure S1: Normal distribution of tensile strength and fracture toughness of *Pinus sylvestris var. mongolica Litv*. with the ratio of latewood zone with less than 17% (a), 17~22% (c) and more than 22% (g) growth rings based on BEM solution.
