# Supplementary material for: Study on the Fracture Toughness of Softwood and Hardwood Estimated by Boundary Effect Model
Source: Materials (Basel). 2022 Jun 6;15(11):4039. doi: 10.3390/ma15114039 (PMC9182387; doi:10.3390/ma15114039)
Supplement: Supplementary file 1 [file materials-15-04039-s001.zip › materials-1732291-supplementary.pdf]

Supporting material

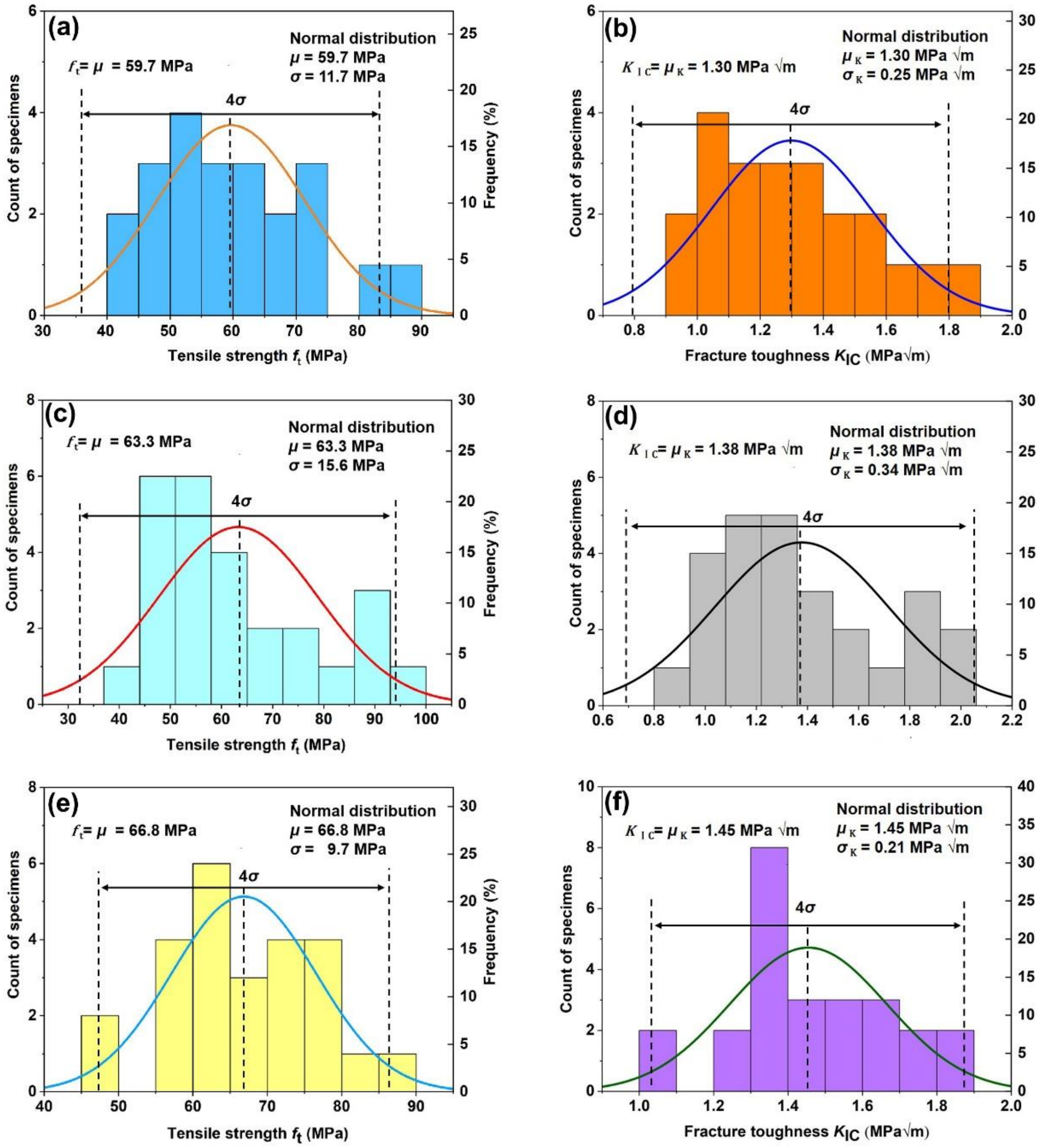

Figure S1. Normal distribution of tensile strength and fracture toughness of Pinus sylvestris var. mongolica Litv. with the ratio of latewood zone with less than 17% (a), 17~22% (c) and more than 22% (g) growth rings based on BEM solution.
